# COVID-19: Not a thrombotic disease but a thromboinflammatory disease

**DOI:** 10.48101/ujms.v129.9863

**Published:** 2024-01-22

**Authors:** Shu He, Margareta Blombäck, Håkan Wallén

**Affiliations:** a Department of Clinical Sciences, Danderyd Hospital, Karolinska Institutet, Stockholm, Sweden; b Division of Coagulation Research, Department of Molecular Medicine and Surgery, Karolinska Institutet, Stockholm, Sweden

**Keywords:** COVID-19, endothelia damage, hyper-coagulation, platelet activation, fibrinolysis shutdown

## Abstract

While Coronavirus Disease in 2019 (COVID-19) may no longer be classified as a global public health emergency, it still poses a significant risk at least due to its association with thrombotic events. This study aims to reaffirm our previous hypothesis that COVID-19 is fundamentally a thrombotic disease. To accomplish this, we have undertaken an extensive literature review focused on assessing the comprehensive impact of COVID-19 on the entire hemostatic system. Our analysis revealed that severe acute respiratory syndrome coronavirus 2 (SARS-CoV-2) infection significantly enhances the initiation of thrombin generation. However, it is noteworthy that the thrombin generation may be modulated by specific anticoagulants present in patients’ plasma. Consequently, higher levels of fibrinogen appear to play a more pivotal role in promoting coagulation in COVID-19, as opposed to thrombin generation. Furthermore, the viral infection can stimulate platelet activation either through widespread dissemination from the lungs to other organs or localized effects on platelets themselves. An imbalance between Von Willebrand Factor (VWF) and ADAMTS-13 also contributes to an exaggerated platelet response in this disease, in addition to elevated D-dimer levels, coupled with a significant increase in fibrin viscoelasticity. This paradoxical phenotype has been identified as ‘fibrinolysis shutdown’. To clarify the pathogenesis underlying these hemostatic disorders in COVID-19, we also examined published data, tracing the reaction process of relevant proteins and cells, from ACE2-dependent viral invasion, through induced tissue inflammation, endothelial injury, and innate immune responses, to occurrence of thrombotic events. We therefrom understand that COVID-19 should no longer be viewed as a thrombotic disease solely based on abnormalities in fibrin clot formation and proteolysis. Instead, it should be regarded as a thromboinflammatory disorder, incorporating both classical elements of cellular inflammation and their intricate interactions with the specific coagulopathy.

## Introduction

In December 2019, Coronavirus Disease in 2019 (COVID-19), caused by the infection of severe acute respiratory syndrome coronavirus 2 (SARS-CoV-2), quickly became a major public health concern (1). Initially, its specific pathogenesis was unclear. As a growing number of patients developed pulmonary embolism (PE), however a life-threatening complication (2–4), thrombotic events were thought to be responsible for the high fatality rate. Numerous autopsy studies were conducted to test this hypothesis. For example, Wichmann et al. examined 12 patients who died with a polymerase chain reaction-confirmed diagnosis of COVID-19 (5). Visual inspections revealed that 7 out of 12 patients (58%) had deep vein thrombosis (DVT) in their legs, while PE was identified as the direct cause of death in 4 of the fatal cases. These autopsy specimens were also analyzed using histopathological methods, which showed the presence of microvascular thromboembolism in lung tissue. Similar postmortem investigations have been conducted by numerous other researchers, revealing various types of thrombotic events in different locations, predominantly in the lungs and occasionally in certain extra-pulmonary organs (6–14).

Extensive research has been conducted to investigate the potential mechanisms behind thrombotic events in COVID-19. Most authors generally concur that the viral infection amplifies the body’s blood clotting properties and activates platelets, while suppressing the breakdown of blood clots (15–20). Therefore, as scholars in the field of hematology, our initial hypothesis centered on COVID-19 being a thrombotic disease. In order to gain a deeper understanding of the disease’s origins and progression, thereby reinforcing our initial hypothesis, we embarked on an extensive review of the existing literature.

Before proceeding to the description later, it should be noted that COVID-19 patients included in this review were mostly severe cases; the control groups were composed of healthy individuals, normal donors, or cases with other acute respiratory distress syndromes (ADSs).

## Key components of COVID-19 pathogenesis

### Viral infection and inflammation

Once SARS-CoV-2 enters the airway, it penetrates lung epithelial cells or pulmonary vascular endothelial cells, via binding its spike protein to a host receptor, mainly to be angiotensin-converting enzyme 2 (ACE2) exposed on the membrane of target cells (21, 22). When the body’s immune cells detect the viral infection, they release cytokines and chemokines (inflammatory modulators, IMs) to signal other immune cells to fight the infection and, meanwhile, to attack patient’s own tissues/organs (23). Several published studies have demonstrated increased plasma levels of IMs in patients with COVID-19 (24–29). For instance, Tufa et al. conducted a study measuring plasma concentrations of 39 IMs and found that more than 70% of them were higher in COVID-19 cases (*n* = 126) than in healthy controls (*n* = 134) (*P* < 0.05 – 0.0001) (29).

### Endothelia damage

Ackemann et al. applied an immuno-histochemical method to identify ACE2 in postmortem lungs. They observed that COVID-19 cases had increased counts of ACE2-positive signals when compared to uninfected individuals, together with higher expression in endothelial cells than in alveolar epithelial cells (30). Thus, some tissues are more vulnerable to SARS-CoV-2 infection due to increased ACE2 expression. The higher ACE2 expression in endothelial cells than in alveolar epithelial cells indicates the vascular endothelium to be an ACE2-rich tissue that is inevitably damaged by the virus. This idea has been proven by numerous evaluations made on pulmonary autopsy specimens from COVID-19 patients (31–35). For example, Ackmann et al. found interstitial and perivascular lymphocytic pneumonia with multifocal endothelitis (30), while Copin et al. identified cytoplasmic vacuolization and cell detachment in small- to medium-sized pulmonary arteries (36). Multiple hemostatic-related components are normally synthesized/stored in endothelial cells. Following SARS-CoV-2 infection, they were overexpressed or elevated in circulation, plausibly validating increase in cellular filterability due to endothelia damage (37–42).

## Impact of COVID-19 on hemostatic system

### Blood coagulation

#### Initiation of coagulation

Tissue factor (TF) is a crucial component in initiating the extrinsic coagulation system. However, due to the lack of international TF standards, measuring plasma levels of TF antigen is challenging. To address this limitation, alternative methods have been employed by researchers. For example, Rosell et al. measured the circulating levels of TF-positive extracellular vesicles (TF-EVs) in 100 cases with COVID-19 and 28 healthy controls (43). Multiple groups performed similar investigations and also found evidence of an increase in TF-EVs associated with the presence of COVID-19 (44–47). Additionally, Subramanian et al. applied fluorescent reagents to detect TF-RNA in postmortem lung tissues from three subject groups: (1) SARS-CoV-2-infected patients who died of ARDs, (2) non-infected patients who died of other ARDs, and (3) non-infected patients who died of other diseases such as cancer. The authors found that signals of TF-RNA in group A were 2.1-fold higher than in group B (*P* < 0.01) and 11-fold higher than in group C (*P* < 0.001) (48). Likewise, Girard et al. determined RNA sequencing in peripheral blood mononuclear cells, revealing a 5.2-fold enhancement in TF transcript expression for patients with severe COVID-19 compared to those with mild or moderate disease (*P* < 0.05) (49). In addition, Martinelli et al. reported that the complex of activated Factor VIIa (FVIIa) and antithrombin was elevated in COVID-19 pneumonia (50). This finding suggests a potential over-expression of TF indirectly because antithrombin specifically interacts with FVIIa only when FVIIa is bound to TF (51, 52).

There have been published data, suggesting that the viral infection promotes the activation of the intrinsic system, as demonstrated in the following examples. Henderson et al. identified that in comparison with healthy individuals, COVID-19 patients had elevated levels of protease-serpin complexes, such as complexes of FXIIa:C1-INH, kallikrein:C1-INH, FXI:C1-INH, and FXIa:α-antitrypin, along with higher levels of single proteases, such as FXI and cleaved/non-cleaved high molecular weight kininogen (45). Additionally, a western blot assay made by Wygrecka et al. revealed that free FXII molecules were significantly decreased in plasma samples from COVID-19 patients (53). This outcome suggests excessive consumption of FXII when the intrinsic system progresses further in the disease, consistent with previous findings in other severe illness (54, 55).

#### Thrombin generation

Theoretically, the increased activation of extrinsic and intrinsic systems by SARS-CoV-2 infection should bring up the activities of FIXa, FXa, and thrombin (56). As a proponent of this, Henderson et al. (45) and Busch et al. (57) reported elevated levels of FIX activity and FX activity, as well as complex of FIXa:antithrombin in patients with COVID-19. Furthermore, multiple groups found elevated circulating complexes of thrombin:antithrombin (TAT), implicating that this illness can boost thrombin generation through activation of the common coagulation pathway (45, 57–59).

The Calibrated Automated Thrombin Generation (CAT) assay is a fluorogenic-based method that allows for the quantification of endogenous thrombin in clotting plasma (60). In quite a few cases with SARS-CoV-2 pneumonia, testing results by this approach were not consistent with the anticipated increase of thrombin generation. For instance, most studies demonstrated that the patients had delayed ‘Lag Time’ (time until thrombin is generated) and/or unchanged ‘Time to Peak Thrombin’ (time needed to reach the peak level of generated thrombin) (42, 61–69). Furthermore, only a subset of studies reported increases in ‘Peak Thrombin’, ‘Endogenous Thrombin Potential’ (ETP), and thrombin generation ‘Velocity’ (42–69). These unexpected observations in the CAT assay may be attributed to elevated plasma levels of anticoagulant factors, such as thrombomodulin (TM) – a tissue factor pathway inhibitor (TFPI), existing in certain COVID-19 patients, as shown by published data (41, 42, 68, 70). Moreover, it is crucial to conduct further evaluations to determine the reliability of using Coagulation Assays (CAT) for testing COVID-19 samples that potentially contain antiphospholipid antibodies (aPLs). CAT is a well-known phospholipid-dependent approach, and the presence of aPLs in the samples may influence the accuracy of the assay results (71–73).

### Fibrin formation

Once thrombin is generated, the process of blood coagulation has reached its final step: thrombin activates fibrinogen to form fibrin monomers, which then polymerize into a network structure (74). Under normal conditions, the produced fibrin is maintained at normal levels and used for wound healing or hemostasis. In acute states of trauma or inflammation, the liver responds by synthesizing as much fibrinogen as possible to repair severe damage to cells/tissues (75). Therefore, fibrinogen hyperplasia, as evidenced by an increase in plasma levels of fibrinogen antigen/activity, is frequently shown in patients with COVID-19 (42, 45, 53, 58, 61, 75–79).

As fibrinogen is the major substrate for thrombin – the key coagulating protease, increased circulating concentrations of fibrinogen can result in increased fibrin formation, even without an obvious enhancement in the thrombin generation potential. For example, Bouck et al. employed a turbidity method to assess the fibrin-generation kinetics in plasma samples. Despite the ‘Lag time’ and the ‘Time to reach plateau of fibrin turbidity’ to be prolonged and unchanged, respectively, the assay still presented significant elevation in ‘Turbidity change’ (higher turbidity reflecting greater amounts of fibrin to be formed) in COVID-19 cases when compared with healthy donors (42). Moreover, using a laser scanning confocal microscopy or a scanning electronic microscope, Wvgrecka et al. reported that the fibrin network became noticeably tighter in COVID-19 patients than in normal controls, while the fibrin network porosity was also decreased by higher fibrinogen concentrations (53).

Since the first article on COVID-19, numerous authors have reported significant increases in D-dimer levels in this disease (45, 55, 66, 78, 78, 80, 81). D-dimer is the end product of fibrin proteolysis; its plasma levels are dependent on the quantity of fibrinogen/fibrin in the circulation. Therefore, the significantly elevated circulating D-dimer concentration in COVID-19 can serve as an important indicator for hyper-turnover of fibrinogen and fibrin.

### Platelet activation

#### Hyper-activation of platelet

Von Willebrand Factor (VWF), platelet factor 4, soluble P-selectin, soluble C-type lectin-like receptor 2, etc. are regularly stored in platelet granules or expressed on platelet membrane. When platelet activation occurs, they would be released into bloodstream, causing their plasma levels to rise. In the context of COVID-19, heightening in these proteins’ concentrations has been reported by many studies, which are considered biomarkers of platelet hyper-activation following the viral infection (16, 82).

According to a review conducted by Iba et al., a meta-analysis of nine studies revealed that some patients with severe SARS-CoV-2 pneumonia exhibited a moderate decrease in platelet count, a condition referred to as thrombocytopenia (83). This finding has also been reported by other authors (16, 84). In COVID-19, thrombocytopenia is often accompanied by an increase in mean platelet volumes (MPV), which serves as a signal of platelet proliferation. The increased MPV suggests that the decrease in platelet counts is likely a result of excessive consumption associated with increased formation of platelet plugs, rather than dysfunction in the bone marrow. Moreover, it is understood that the increased MPV favors platelet aggregation and fibrinogen binding (16, 83, 84).

#### Mechanisms that enhance platelet activation

There are at least three distinct mechanisms that have been defined to elevate platelet activation in COVID-19.

The first mechanism is related to the inflammatory response to SARS-CoV-2 infection and the resulted cytokines storm. Many of the released IMs are known to trigger or amplify platelet activation; IL-6, IL-1β, and tumor necrosis factor (TNF)α are particularly important in this regard (16, 84–87). Furthermore, the disease-derived inflammation can stimulate platelets to express TF, thereby contributing to interaction between platelets and monocytes to form more pro-coagulating platelet-monocyte aggregates.

The second mechanism is related to SARS-CoV-2 itself. There have been observations, indicating that the virus can directly invade platelets. The exact receptor on the platelet membrane to which the virus spike protein binds remains unknown, but ACE2 is a potential candidate receptor for this interaction (87–89). For instance, in a study by Manne et al., RNA sequencing was used to analyze platelets from COVID-19 cases. The authors found various changes of gene expression in pathways associated with protein ubiquitination, antigen presentation, and mitochontrial dysfunction (89). Surprisingly, no mRNA for ACE2 was observed, but the integration of the viral mRNA gene into the platelet gene sequence was detected. This indicates that the platelet can take up SARS-CoV-2 mRNA independent of ACE2 (89). The genetic changes mentioned earlier appear to underlie the pathology of enhanced platelet activation, since the same samples also displayed elevated circulating aggregates of platelet-neutrophils, monocytes, and T cells, along with increased spread of platelets on fibrinogen and collagen (89).

The third mechanism is related to an imbalance between VWF and ADAMTS-13, which results in elevations in large-sized VWF multimeres with ensuing platelet-rich microthrombosis. VWF is a large adhesive multimeric (up to 20.000 kDa) involved in platelet adhesion in a size-dependent manner (90, 91). ADAMTS-13 is the main VWF cleavage protease responsible for maintaining the appropriate size of VWF molecules (92, 93). Congenital or acquired defects in ADAMTS-13 are associated with enhancement in large VWF multimers, bringing about the formation of platelet-rich plugs and systemic microthrombosis, a condition called thrombotic thrombocytopenic purpura (TTP) (94). With respect to COVID-19, many studies have reported that the patients are in high risk for TTP (95–97). Initially, the SARS-CoV-2-induced TTP was identified according to the clinical manifestations of microthrombosis and laboratory findings of reduced ADAMTS-13 (98). However, TTP’s clinical features may appear in some COVID-19 cases with normal or slight decrease of ADAMTS-13 levels. To increase the understanding and avoid under-diagnosis of this life-threatening complication, an assessment of VWF/ADAMTS-13 ratio has recently been employed (99, 100). Based on this, an elevated VWF/ADAMTS-13 ratio may indicate ‘acquired ADAMTS-13 deficiency’, which retains larger circulating VWF molecules to increase platelet binding and risk of microthrombosis. A review on 20 studies (encompassing 1,197 COVID-19 cases) by Favaloro et al. demonstrated that VWF plasma levels were typically elevated in all patients, while ADAMTS-13 levels were either normal or reduced (101). As a result, the values of VWF/ADAMTS-13 ratio consistently exceeded the normal range and demonstrated a positive correlation with the severity of thrombotic events. Further assessments may be required to determine whether the potential value of this ratio can be used as a clinical tool for estimating high risk of TTP in COVID-19.

### Fibrinolysis

#### Individual pro-fibrinolytic and antifibrinolytic components

According to a study conducted by Henry et al., circulating plasminogen levels in COVID-19 patients upon admission were not abnormal. However, a slight but significant downward trend in plasminogen levels was shown with increasing clinical severity, probably due to consumption during the breakdown of clots (102). In addition, several published articles have indicated elevated levels of another quantitative marker of plasminogen, that is, the complex of plasminogen: α2-antiplasmin, as well as pro-fibrinolytic components (e.g. plasminogen activators: t-PA and u-PA) and antifibrinolytic components (e.g. plasminogen activator inhibitor (PAI)-1, α2-PI, and TAFI, see the abbreviations) in patients with COVID-19 (40, 41, 65, 103–105). It is important to note that since the aforementioned data only reflect altered concentrations or functions of each individual variable, their collective impact of these changes on fibrin digestion remains unknown.

#### D-dimer information and global fibrinolysis potential

In addition to being considered an important indicator for hyper-turnover of fibrinogen and fibrin as earlier mentioned in this article, increased D-dimer levels in COVID-19 mean that there should be a supra-normal degree of fibrin degradation taking place somewhere in the patients. Contrary to this initial expectation, however, several assessments presented contrasting findings and perspectives when using either thromboelastography (TEG) or rotational thromboelastography (ROTEM) methods to examine COVID-19 patients. In the viscoelastic tests, coagulation can, for example, be initiated by adding TF or kaolin/ellagic acid, and the fibrinolysis is triggered by residual tPA (approach 1) or rtPA added (approach 2) (18). For example, Pavoni et al. utilized approach 1 to observe a shorter Clot Formation Time (CFT), higher Max Clot Firmness (MCF), and lower Max Lysis (ML) in the majority of the patients compared to the reference ranges (106). In another study which also ran approach 1, Salem et al. reported that among 52 COVID-19 patients, 31% exhibited increased MCF values, while all patients displayed significantly reduced Lysis at 30 min (LY30) (107). Employing approach 2, Collett et al. found raised clot firmness at 10 min after clotting time (10A) and elevated MCF, along with minimal ML in all COVID-19 cases (108). Furthermore, Bachler et al. employed approach 2 to detect increased MCF, decreased ML, and delayed time required to reach 50% MCF (LT) in 20 COVID-19 patients when compared to 60 healthy individuals (109). On the whole, the aforementioned surveillances, together with others (20, 110–114), consistently designate hyper-coagulation in COVID-19, which led to the formation of fibrin clots highly resistant to the effects of endogenous plasmin.

Thus, increased D-dimer levels and decreased clot proteolysis, as assessed with viscoelastic methods, are, indeed, present concurrently in cases suffering from SARS-CoV-2 pneumonia. Some authors have referred to this paradoxical combination as ‘fibrinolysis shutdown’ (20, 110–114).

#### Understanding ‘Fibrinolysis Shutdown’

The term ‘fibrinolysis shutdown’ has traditionally been used in earlier published documents to describe changes in fibrinolysis following severe trauma (115–118). In cases of severe trauma, the initial hemostatic response to tissue damage trends to causing thrombin generation and fibrinogen polymerization. The cleaved fibrinogen molecules serve as binding sites for both plasminogen and t-PA, which facilitates the activation of plasminogen into its active form – plasmin. Then, plasmin acts upon fibrin, breaking it down into smaller fragments, including D-dimers. Typically, after severe trauma, the highest levels of plasma D-dimer can be detected approximately 3 h later (117). Once plasminogen is activated, the liver rapidly removes t-PA from circulation and starts to synthesize fibrinolysis inhibitors, primarily PAI-1. Around 6 h after the injury, plasma concentrations of PAI-1 reach their peak levels and then return to the normal within 1 day for cases with good outcomes or remain elevated for 2–3 days for cases with poor outcomes (117). That is, in cases of trauma with heightened coagulation, a distinct temporal pathological sequence occurs. Initially, fibrinolysis is triggered but is subsequently countered and collapses due to the hyper-action of PAI-1 (115–118).

Despite the fact that the recently published studies have led to the term ‘fibrinolytic shutdown’ being applied to name the impaired fibrinolytic activities in COVID-19, the available data in the literature remain limited, which do not provide sufficient insights into the underlying pathological mechanisms (20, 110–114). To bridge this knowledge gap, it is crucial to gather additional data through laboratory and clinical investigations. Ideally, blood samples would be collected from COVID-19 patients at various time points post onset of the disease. By determining the fibrinolytic capacity using the viscoelastic methods and comparing it with changes in plasma levels of D-dimer, t-PA, and PAI-1, researchers may potentially uncover the expected pattern of fibrinolysis being ‘turned on’ and subsequently ‘turned off’.

Besides, when investigating fibrinolysis in the context of COVID-19, a common question that arises is about the seemingly limited ability of increased t-PA levels to counteract the antifibrinolytic effect caused by PAI-1. A study conducted by Chandler et al. sheds light on this phenomenon. The authors used a two-compartment model to analyze the hepatic clearance fractions of both active t-PA and t-PA/PAI-1 complexes (PAI-1 is regularly present in a complex with t-PA in plasma). The results revealed that the average hepatic clearance fraction for active t-PA was significantly higher than that for t-PA/PAI-1 complex, at 89 ± 10% with a half-life of 2.4 ± 0.3 min and 48 ± 17% with a half-life of 5.0 ± 1.8 min, *P* = 0.0006 (119). This explanation is further supported by the findings of Whyte et al., that is, in COVID-19, despite both PAI-1 and t-PA levels to be elevated, the clot lysis time (LT) values were only correlated with PAI-1 levels (*r* = 0.68, *P* < 0.001) but not with t-PA levels (103).

#### An alternative hypothesis

Since the initial tissue damage caused by SARS-CoV-2 infection primarily affects the alveolar cells and endothelium of the lungs (120). Some authors have proposed an alternative hypothesis to interpret the simultaneous occurrence of elevated D-dimer levels and reduced clot proteolysis capacity in COVID-19 patients. According to Ibanez et al. and Kwaan et al., tissue damage in the lungs triggers a pathological sequence where fibrinolysis follows a state of hyper-coagulation, facilitated by the activation of plasminogen with the assistance of urokinase-type plasminogen activator (u-PA). As a result of this process, D-dimers are generated and enter the systemic circulation, leading to elevated concentrations in plasma. Concurrently, the acute pulmonary inflammatory response may induce the release of inflammatory cytokines, such as IL-1, IL-6, and IL-17A, which, in turn, upregulate the production of PAI-1. Besides, the damaged alveolar cells may result in decreased surfactant levels and reduced activation of the 53 pathway, further amplifying PAI-1 production. The generated PAI-1 may consequently be accumulated in the lungs or enter the bloodstream, resulting in the inhibition of fibrinolysis either locally or systemically (120–124).

## Thromboinflammation in COVID-19

### Understanding pathogenesis of the coagulopathy

As we stated in the preceding chapters, the virus initially infiltrates the alveolar epithelium and pulmonary vascular endothelium through an ACE2 receptor-dependent mechanism (21, 22). Afterward, a dynamic and multifaceted process unfolds, marked by an exaggerated release of pro-inflammatory cytokines, often termed the ‘cytokine storm’ (23–29). The virus targets multiple types of cells but most critically targets the vascular endothelium. The structural integrity and functionality of endothelium are hence impaired, suggesting viral endothelialitis-induced endothelial injury. In response to these events, the damaged endothelial cells and other activated or infected cells release various pro- and anticoagulants (e.g. TF, VWF, Factor VIII, TM, TFPI, PAI-1, tPA, P-selectin, etc.) (37–42) and inflammatory modulators (e.g. IL-1, IL-6, IL-17A, etc.) (24–29). Many of the released components play pivotal roles in platelet activation while simultaneously influencing coagulation and fibrinolysis.

Apart from the SARS-CoV-2 driven inflammation and endothelial injury, the activated innate immune system also involved, exhibiting distinct responses to the hemostatic system. Here are three illustrative instances related to this topic:

- ***Activation of the Contact/Kallikrein/Kinin System*:** This intricate cascade of enzymatic reactions involves key players such as factor XII, prekallikrein, and high molecular weight kininogen. Except for contributing to thrombin generation, which has been mentioned in the above chapter (51–55), the contact/kallikrein/kinin system can initiate a sequence of events, which releases bradykinin. In COVID-19, elevated levels of bradykinin and its metabolites interact with G protein-coupled receptors, to bring about vasoactive effects like increased vasodilation and vascular permeability. This process may amplify oxidative stress, cytokine release, and the release of procoagulant molecules, creating a pro-thrombotic environment (124–126).
- ***Activation of the Lectin Pathway of the Complement System*:** The lectin pathway, particularly involving Mannose-Binding Lectin (MBL), acts as a pattern recognition glycoprotein, targeting pathogen-associated molecular patterns (PAMPs) on viral surfaces, including SARS-CoV-2. MBL binding to these PAMPs initiates the lectin pathway, activating complement proteins. This process leads to the formation of the C3 convertase enzyme, triggering a cascade that generates anaphylatoxins and membrane attack complexes. Literature data suggest that SARS-CoV-2 infection may enhance the activation of lectin pathway, which potentially contributes to inflammation, endothelial damage, and a pro-thrombotic environment, signaling adverse outcomes in affected patients, such as those experiencing disseminated intravascular coagulation (DIC) (127, 128).
- ***Involvement of Polymorphonuclear Leukocytes and Neutrophil Extracellular Traps*:** Polymorphonuclear leukocytes, a subset of white blood cells, play crucial roles in the innate immune system. One of their important functions is the generation of neutrophil extracellular traps (NETs). NETs are extracellular webs of chromatin, microbicidal proteins, and oxidant enzymes, which are released by neutrophils to contain infections. Evidence from published studies has shown the presence of markers associated with the formation of Neutrophil Extracellular Traps (NETs) in lung specimens from COVID-19 victims, as well as in the sera and tracheal aspirates of COVID-19 patients. These markers embrace heightened levels of circulating DNA and increased neutrophil elastase activity and myeloperoxidase DNA complexes, among others. The enhanced formation of NETs may serve as the pathogenesis of various disorders seen in COVID-19. Particularly, it appears to reinforce the connection of inflammation and endothelial injury with thrombosis, that is within the context explored in this article (129–132).

Indeed, there are additional innate immune responses to SARS-CoV-2 infection beyond what we have described here, as summarized by researchers (133). These responses, along with the further developed inflammation and endothelial injury, underscore the complexity of COVID-19-associated coagulopathy, resulting in occurrence of thrombotic events. Therefore, we consider that COVID-19 should no longer be viewed as a thrombotic disease solely based on abnormalities in fibrin clot formation and proteolysis, but rather as a thromboinflammatory disease with coagulopathy (134–136).

### Development of thromboinflammation across different clinical phases of disease

It has been proposed that SARS-CoV-2 pneumonia can be divided into three main phases (137). These clinical phases span from mild or asymptomatic to moderate and eventually advance to severe cases characterized by the gradual development of ARDs and multi-organ failure.

In *the initially infection phase*, the virus infiltrates the lung parenchyma and begins to proliferate. The virus infiltrates the lung parenchyma, beginning to proliferate. This stage marks the initial response driven by the innate immune system, including ACE2 and monocytes/macrophages, often presenting with mild constitutional symptoms. In the *pulmonary phase*, the inflammatory response featuring vasodilation, increased endothelial permeability, and leukocyte recruitment results in lung injury and hypoxemia, clinically manifesting as ARDs in some patients. Simultaneously, the coagulation system becomes rapidly activated, leading to abnormalities in routine hemostatic tests. These abnormalities encompass heightened platelet aggregation, slight prolongation of APTT and PT, mild thrombocytopenia, and elevated plasma levels of TF-EVs, FVIII, VWF, TAT, fibrinogen, D-dimer, and PAI-1, among others. Furthermore, micro- and/or macro-thrombosis, especially in the lungs, may be observed. In the final phase, known as the *hyper-inflammation phase*, the disease transforms into systemic inflammation, even with reduced viral load. This stage witnesses increased production of various cytokines, including IL-6, IL-2, IL-7, TNF-α, IP-10, MCP-1, MIP-1α, G-CSF, C-reactive protein, lactate dehydrogenase, and more. Acute pulmonary thrombosis, ischemic stroke, myocardial infarction, systemic arterial or venous thrombosis, and multiple-organ failure may occur (137).

The three clinical phases of COVID-19 progress in tandem with the development of thromboinflammation throughout the entire disease course. This observation leads us to consider that, in comparison to other viral infections like the 2002–2003 Severe Acute Respiratory Syndrome (SARS) and Middle East Respiratory Syndrome (MERS), the relatively higher thrombotic risk associated with SARS-CoV-2 pneumonia (138) may be explained by the stronger connection between virally driven inflammatory-immune response and damage to the hemostatic system.

## Summary

**Figure 1:**
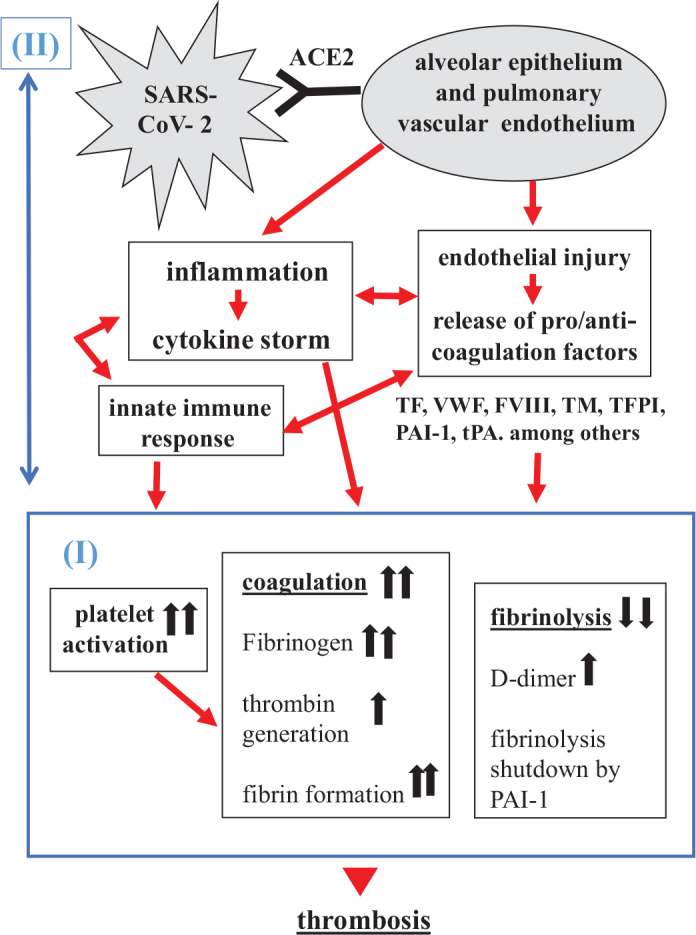
Thromboinflammation – the pathogenesis of hemostatic disorders in COVID-19. One arrow represents increase; two arrows represent greater increase. For more details, see ‘Summary’.

This literature review has yielded the concluding remarks, which are summarized in an illustration (see Figure 1). As represented in part I (below), the hemostatic system in COVID-19 is altered, showing increased platelet activation and coagulation, accompanied by decreased fibrinolysis. It appears that elevated levels of fibrinogen have a more pronounced effect on fibrin formation than an elevated potential of thrombin generation due the existence of specific anticoagulants in plasma. Fibrinolysis is, indeed, initiated following fibrin formation, since heightened D-dimer levels have been detected. However, ‘fibrinolysis shutdown’ happens rapidly due to the overproduction of PAI-1, thereby further amplifying the severity of hyper-coagulation in this disease. In Part II (above), a sequential cascade of events is delineated, starting with ACE2-dependent viral invasion and progressing through induced tissue inflammation, endothelial injury, and innate immune responses. These processes collectively contribute to the coagulopathy described in part I. Given the close interplay between the key players in inflammation and thrombosis, it is appropriate to characterize the pathogenesis of the pro-thrombotic condition in SARS-CoV-2 pneumonia as thromboinflammation.
